# Description of day case costs and tariffs of cataract surgery from a sample of nine European countries

**DOI:** 10.1186/s12962-022-00346-3

**Published:** 2022-03-05

**Authors:** Antonio Olry de Labry Lima, Zuzana Špacírová, Jaime Espín

**Affiliations:** 1 Escuela Andaluza de Salud Pública (EASP), Granada, Spain; 2 CIBER en Epidemiología y Salud Pública (CIBERESP), Madrid, Spain; 3 Instituto de Investigación Biosanitaria Ibs. Granada. Hospitales Universitarios de Granada/Universidad de Granada, Granada, Spain

**Keywords:** Cataract, Costing methodology, Transparency, Economic evaluation

## Abstract

**Background:**

The lack of transparency in the methodology of unit cost estimation and the usage of confidential or undisclosed information prevents cost comparisons and makes the transferability of the results across countries difficult. The objective of this article is to compare the methodologies used in the estimation of the cost of a day case cataract extirpation that are described in the official and publicly available sources and to study how these translate into different unit cost estimates.

**Methods:**

A literature review was conducted to identify the main sources of unit costs of cataract extirpation. A semi-structured questionnaire to obtain information on national costing methodologies was developed and sent to consortium partners in nine European countries. Additionally, publicly available sources of unit cost of cataract surgery in those countries included in the European Healthcare and Social Cost Database (EU HCSCD) were analysed.

**Results:**

The findings showed a considerable diversity across countries on unit costs varying from 432.5€ in Poland (minor degree of severity) to 3411.96€ in Portugal (major degree of severity). In addition, differences were found in the year of cost publication and on the level of detail of different types of cataract surgery. The unit of activity were Diagnosis-Related Groups in all countries except Slovenia. All unit costs include direct costs and variable overheads (except Germany where nursing costs are financed separately). Differences were identified in the type of fixed overheads included in unit costs. Methodological documents explaining the identification, measurement and evaluation of resources included in the unit costs, as well as use of appropriate cost drivers are publicly available only in England, Portugal and Sweden.

**Conclusions:**

We can conclude that while unit costs of cataract extirpation are publicly available, the information on methodological aspects is scarce. This appears to pose a significant problem for cross-country comparisons of costs and transferability of results from one country to another.

**Supplementary Information:**

The online version contains supplementary material available at 10.1186/s12962-022-00346-3.

## Background

Untreated cataracts are the leading cause of blindness worldwide and the second leading cause of visual impairment. It causes the opacity of the eye lens leading to blurred or reduced vision. The only effective intervention is a surgical operation that involves the removal of the blurred lens and subsequent implantation of a lens [1]. Cataract disease and healthcare represent a significant health burden and economic expense, both in direct (healthcare) and indirect (productivity) costs [2]. It is estimated that around 11.9 million people worldwide suffer from impairment problems or blindness caused by glaucoma, diabetic retinopathy and trachoma. Prevention of vision impairment in this population would cost $32.1 billion US dollars [3].

Economic evaluation is an important tool for adoption and reimbursement of health technologies. The lack of transparency in the methodology of unit cost estimation and the usage of confidential or undisclosed information prevent cost comparison, which, in turn, makes the transferability of the results across countries difficult. The European Commission (Horizon 2020) project IMPACT-HTA aimed to understand the variation of costs across European countries. One of the outcomes of this project was the European Healthcare and Social Cost Database (EU HCSCD), a minimum common dataset of international costs (including primary resources, composite goods and services and complex processes and interventions) which can feed into health-economic evaluations and enable transfer of models across countries [4]. All costs included in the EU HCSCD come from official sources of nine European countries. A User’s guide describing the database and methodological aspects of included cost items is available [5].

The objective of this article is to analyse and compare official and publicly available sources of cost of cataract extirpation in nine European countries included in the EU HCSCD.

## Methods

A literature review was carried out consulting PubMed and Scopus databases to identify articles estimating the cost of cataract extirpation that were published after 2005 in English, Spanish and French. This search was completed with an additional search on Google Scholar. The search terms used were: (cataract AND Cost AND “cost analysis" [MeSH] AND Europe). The literature search was verified by a librarian with an extensive experience in the field of public health. The reference lists of relevant studies identified from the search were also reviewed. The objective of the literature review was to identify the sources of costs and/or tariffs of cataract surgery and ensure that there were no additional publicly available sources to those included in the EU HCSCD.

The methodology used to construct DRG costs has been described in some detail by previous EC projects HealthBASKET [6] and EuroDRG [7]. To update these documents and to fill in gaps a semi-structured questionnaire for each of the 9 countries was sent to expert collaborators in the IMPACT-HTA project. The questionnaire was piloted and revised several times after receiving feedback from the cost experts. The final version had 17 items grouped in 8 dimensions (Additional files 1, 2). The questionnaire included a glossary of terminology and examples based on the literature [8] in order to clarify all the concepts used and to avoid errors.

No ethical approval was required since we were not dealing with patient data.

## Results

The literature review retrieved 54 articles; five articles were based on unit costs obtained from publicly available databases. A PRISMA diagram is provided as Additional file 3. One article used the English national tariff database, the French national cost database (ENC), the German national Diagnosis-Related Group (DRG) tariffs and the Italian national DRG tariffs. One article used the French national cost database (ENC). Three articles used the English Reference cost database (Table 1).

**Table 1:** Results of the literature search

Author (year)	Country	Source of cost data
Qatarneh (2012) [9]	UK	The indicative costs of attendances and the various additional procedures were obtained from the Department of Health reference cost guidance using NHS Health Resource Group (HRG) version 4 and 2009 data
Lafuma (2008) [10]	France, Germany and Italy	Publication de *L'échelle Nationale des Coûts (données 2003–2004*). *Agence Technique de l'Information sur l'Hospitalisation*. [http://www.atih.sante.fr/?id=000370000DFF] DRG on Line. [http://www.drg.it] *Medizincontrolling*/DRG Research Group. *Universitätsklinikum Münster Westfälische Wilhelms-Universität Münster*. 2007, [http://drg.uni-muenster.de/de/webgroup/m.brdrg.php?baserate=2900&showgrafik=0&version=GDRG2005&mdc=02]
Pezzullo (2018) [11]	UK	Reference Cost data collected by the Department of Health. For the rest of UK: Scotland’s Health Service Costs, Wales’ Health Statistics Wales, and Northern Ireland’s Reference Costs
Cooper (2015) [12]	UK	The costs of procedures for treating post-surgical complications and consequences were estimated using 2011–12 UK NHS reference costs. Department of Health. NHS Reference Costs 2011/2012 https://www.gov.uk/government/publications/reference-costs-guidance-for-2011-12
Cornut (2013) [13]	France	These costs were compared to each other and to the target costs of the Diagnosis Related Groups for public hospitals (*Groupes Homogènes de Séjours* [GHS]) concerned, extracted from the analytic accounting data of the French National Cost Study (*Étude Nationale des Coûts* [ENC]) for 2009

Analyzed articles

Source: Own elaboration

UK, United Kingdom; NHS, National Health Service; DRG, Diagnosis-Related Group

Table 2 shows information on costs, methodology and resources included in unit cost collected from the IMPACT-HTA project countries: England, France, Germany, Italy, Poland, Portugal, Slovenia, Spain and Sweden. The range of unit costs varied from 432.45€ in Poland (minor degree of severity) to 3411.96 € in Portugal (major degree of severity). Year of cost publication varied from 2012 (Italy) to 2019 (England, France and Sweden). In Portugal, the tariff of inpatient and day case cataract surgery was the same. In all countries, day case cataract surgery refers to an admission to hospital but without an overnight stay and takes place in hospital.

**Table 2:** Description of costs and tariffs of day case cataract surgery

Country	Unit of activity	Unit value	Description of costing item, Monetary value (€)^*^	Year of cost publication	Institution responsible for calculation/publication of cost/tariff	Resources included	Costing methodology
England	DRG	Cost	Complex, Cataract or Lens Procedures, with CC Score 0–1 (1,555€), Very Major, Cataract or Lens Procedures, with CC Score 0–1 (1,168€), Intermediate, Cataract or Lens Procedures, with CC Score 0–1 (991€) [17]	2019	NHS England	All direct costs, variable overheads and fixed overheads (except teaching cost and research cost)	Top-down micro-costing
		Tariff	Complex, Cataract or Lens Procedures, with CC Score 0–1 (1,603€), Very Major, Cataract or Lens Procedures, with CC Score 0–1 (1,040€), Intermediate, Cataract or Lens Procedures, with CC Score 0–1 (829€) [18]	2017	NHS England	Direct costs, variable overheads and fixed overheads (except teaching cost and research cost)	Based on costs
France	DRG	Cost	Interventions on the lens with or without vitrectomy (1,668€) [15]	2017	Scansanté (ATIH database)	Clinical activities (caregivers, medical and clinical staff), medico-technical activities (operating room, anaesthesia), fixed overheads (119€) (logistics and general management (general administrative services, hotel services, management information system, maintenance, restoration)) and variable overheads. Fixed overheads excludes research costs, teaching costs and financial costs	Top-down micro-costing
		Tariff	Interventions on the lens with or without vitrectomy (1,355.87€) [19]	2019	ATIH	N/A	Based on costs
Germany	DRG	Cost	Bilateral extracapsular extraction of the lens with congenital malformation of the lens (2,263€); Extracapsular extraction of the lens without congenital malformation of the lens (1,634€) [20]	2019	InEK	All direct costs, variable overheads (except nursing) and fixed overheads (except teaching cost, research cost, depreciation of building and financial cost)	Top-down micro-costing
Italy	DRG	Tariff	994€ [21]	2012	Italian Ministry of Health	All direct costs, variable overheads and fixed overheads (except teaching cost, research cost and financial cost)	Top-down micro-costing
Poland	DRG	Tariff	Severity 1 (504.99€), severity 2 (432.45€) [22]	2018	National Health Fund	All direct costs, variable overheads and fixed overheads (except teaching cost, research cost and financial cost)	Top-down gross-costing
Portugal	DRG	Tariff	Severity 1 (1313.65€), severity 2 (1771.19 €) and severity 3 (3411.96€) [23]	2018	National Health Service	All direct costs, variable overheads and fixed overheads	Top-down micro-costing
Slovenia	DRG	Cost	593.55€ [24]	2019	Health Insurance Institute of Slovenia	Personnel costs (1.1 specialist, who, in turn, includes 0.1 anaesthesiologist, 1 nurse, 1 health care technician and 0.47 administrative technical worker), variable overheads, premium for additional pension insurance, material, depreciation and additional funding for computerization 2 outpatient examinations (1 before and 1 after surgery). Financial costs and depreciation of building are excluded	Top-down gross-costing
Spain	DRG	Cost	951€ [25]	2017	Spanish National Health System	All direct cost, variable overheads and fixed overheads (except teaching cost, research cost, depreciation of building and financial cost)	Top-down micro/gross costing (depends on hospital)
	^+^	Tariff	^+^	^+^	Autonomous Region Government	^+^	Not clear
Sweden	DRG	Tariff	Unilateral lens surgery (637€ in Blekinge, Halland and Kronoberg region; 695€ in Skåne region; 701€ Jönköpings län, Kalmar län and Östergötland region). Bilateral lens surgery (1,316€ in Blekinge, Halland and Kronoberg region; 1,435€ in Skåne region; 1,441€ in Jönköpings län, Kalmar län and Östergötland region) [26, 27]	2019	Swedish healthcare regions	All direct cost, variable overhead and fixed overhead (except research cost and teaching cost)	Top-down micro-costing

Source: Own elaboration

^*^Costs published in currency different from euros were converted into euros using the European Healthcare and Social Cost Database (EU HCSCD) (https://www.easp.es/Impact-Hta/Default)

^+^This depends on the Autonomous Region

DRG, Diagnosis-Related Group; CC, complexity and comorbidity; NHS, National Health Service; ATIH, *Agence Technique de l’Information sur l’Hospitalization*; InEK*, Institut für das Entgeltsystem im Krankenhaus*

Significant variability in the level of detail of different types of cataract surgery was observed. Thus, there were countries that showed up to 9 different costs depending on type of the procedure or degree of complexity (England), while; other countries published a single cost of the procedure (Germany, Italy, Poland, Slovenia). The unit of activity was DRG in all countries except in Slovenia, where the estimation of cost was based on the breakdown of various cost items (materials and its depreciation, fixed overheads, personnel and extra pays) included in the procedure [14]. The DRG cost was available in Germany and Slovenia, while Italy, Poland, Portugal and Sweden published DRG tariffs. England, France and Spain published both DRG tariff and cost. Additionally, in France, both total unit cost of the DRG and cost of different DRG subheadings (infrastructure cost, personnel cost, logistics and general management, medico-technical activities such as operating room, etc.) were published [15]. In England, a methodological document that classifies each cost element (resource) in direct cost, variable overhead or fixed overhead, as well as specifies of cost drivers used to allocate each cost element to the final cost object was identified [16]. However, the number of units of each resource included in the total cost of the cataract surgery was not specified in the document nor in the document of any other country (only partially in case of Slovenia as can be seen in Table 2). This lack of detail hindered transparency. In Sweden, the tariff depended on whether the patient was treated in a hospital in their home region or another region.

Another important finding relates to the differences between DRG costs and DRG tariffs. In England, each cataract surgery subtype has both DRG costs (referred in England as reference costs) [17] and DRG tariffs [18]. They are published by NHS England and NHS Improvement. DRG costs are based on finished consultant episode (the total time a patient spends in a hospital in the continuous care of one consultant) [28]. In order to produce 2016/2017 DRG costs, all costs and resources from the financial year 2016/2017 (1st year) are collected in 2017/2018 (2nd year) and are analysed in 2018/2019 (3rd year) [29]. DRG tariffs are used for reimbursement and are calculated based on DRG costs [28], though with some important differences DRG tariffs are based on “spells” (total time a patient spends in a hospital on a continuous basis) [28]. Additionally, DRG tariffs contain incentives for providers to prioritize certain types of activity or to increase efficiency as well as other adjustments. In France, DRG costs of cataract surgery are published by the ATIH database ScanSanté from the hospital production point of view and are lower than DRG tariffs. DRG costs are based on costs provided by a sample of public and private hospitals on an annual basis [15, 30]. DRG tariffs associated with the cataract surgery are published by the Technical Agency on Information about Hospitalization (*Agence Technique de l’Information sur l’Hospitalization*; ATIH) from the social health insurance point of view. The health insurer in France does not reimburse the hospital for the full DRG tariff; a proportion of the tariff must be paid by the patient or by an additional insurance [31]. No official document was found that explained how the French DRG costs and DRG tariffs were constructed. Spanish DRG costs are published by the Spanish National Health Service. The most recent version was calculated from a sample of 79 hospitals in 2017 [32]. Spain has a decentralized health system that consists of 17 regions and all of them publish their own tariff list. No official documents were found explaining how the costs or tariffs were calculated.

## Discussion

One of the findings of this study is that detailed documents explaining how the resources are identified, measured and valued, what type of cost drivers were used in estimation of costs and what type of resources were included in calculation of unit costs are missing in most countries except England, Portugal and Sweden. This suggests that there is a lack of transparency in the costing methodology used in estimating costs and setting public prices and/or tariffs for different procedures carried out by health systems. This result is more striking considering that the economic evaluation guidelines of the different countries mention the need for an adequate identification of all resources included in the final unit costs.

Fattore and Torbica [33] compared costs and tariffs of cataract surgery in nine European countries; six of them were included in this study. The data were collected on the basis of vignettes using common cost templates. The average total cost of the procedure was 714€ (sd: 311€); the average cost of lens was 157€ (sd: 57€); the average cost of personnel was 221€ (sd: 151€); the average cost of infrastructure was 178€ (sd: 158€); and the average of other costs were 175€ (sd: 149€) [33]. In this study, the total cost of cataract surgery is broken down into several categories. However, no information on the type and number of resources included in the total cost (e.g., the type of healthcare professionals included in the average cost of personnel) has been provided.

The results of the study described in this article demonstrate the need for the authorities of the European countries to include detailed information on the estimation of the costs/tariffs in the official sources, which can ensure the transferability of the results across countries. Geissler et al. (2015) mentions the idea of ‘a common European DRG system to define homogeneous groups of patients across different countries’ [34]. This would enable a reliable comparison of costs across countries in that if the resources that compound a cataract surgery were the same in all the countries, the differences in total costs would be due to differences in unit costs of the resources. However, the existing need entails providing detailed information on total costs of the procedure. It should be highlighted that by having detailed information on costs we mean that the type and the number of units of each resource included in the final cost object as well as the method used in resource identification, measurement and valuation should be well described and publicly available.

The result of standardized economic evaluation is that it would be transferable from country A to country B without having to develop the same economic model from scratch in country B.

The study has several limitations. First, the year of cost publication varies among countries, so the costs do not refer to the same year. However, costs inflated to 2019 prices using both Consumer Price Index and Gross Domestic Product can be found at the EU HCSCD webpage [4]. Second, the non-existence of publicly available costing documents describing in detail the type and number of resources included in unit cost would be an important source of non-comparability and non-transferability of results from one country to another.

## Conclusions

This study highlights the need of methodological documents describing the resources included in the estimation of unit costs to be publicly available. Enhancing transparency in and accessibility of methodological costing documents will improve the transferability of economic evaluations across countries.

## Supplementary Information

**Additional file 1.** Questionnaire General.

**Additional file 2.** Responses to questionnaire.

**Additional file 3.** PRISMA flow diagram.

## Data Availability

All unit costs and costing documents mentioned in the manuscript are available at https://www.easp.es/Impact-Hta/Default.

## Abbreviations

DRG: Diagnosis-Related Groups
NHS: National Health Service
ATIH: Agence Technique de l’Information sur l’Hospitalization
EU HCSCD: European Healthcare and Social Cost Database
