# Supplementary material for: Description of day case costs and tariffs of cataract surgery from a sample of nine European countries
Source: Cost Eff Resour Alloc. 2022 Mar 5;20:11. doi: 10.1186/s12962-022-00346-3 (PMC8898401; doi:10.1186/s12962-022-00346-3)
Supplement: Supplementary file 1 — Additional file 1. Questionnaire General. [file 12962_2022_346_MOESM1_ESM.docx]

**Survey for Official costing methodology of health care services used to derive unit health care costs for economic evaluation analyses**

**QUESTIONNAIRE INSTRUCTIONS**

This questionnaire aims to obtain information about the cost accounting methods used in your country to calculate the cost of health care services on which the unit costs in economic evaluation analyses are usually based. It would also be helpful if you could indicate the source or internet address of any documentation that would provide further detail for each question. Please, find enclosed a glossary of terms used in the questionnaire at the end of this document.

Please, send the completed survey to Jaime Espín ([jaime.espin.easp@juntadeandalucia.es](mailto:jaime.espin.easp@juntadeandalucia.es)) and Zuzana Špacírová ([zuzana.spacirova.easp@juntadeandalucia.es](mailto:zuzana.spacirova.easp@juntadeandalucia.es)) by May 3rd, 2019. If you have any questions, please do not hesitate to contact us via email or by telephone +34600160806.

**Thank you for your timely response and participation.**

| **Responder Information** |
| --- |
| **Country:** |
| **Institution:** |
|  |
| **Name:** |
| **Email:** |
| **Position:** |
| **Department:** |
| **Telephone:** |

1. **Is there in your country one or several official or frequently used sources of unit costs for economic evaluation?** Please list them and provide access link if available.
2. **Are the unit costs in the former databases based on accounting costs of public or private health care institutions or on other types of monetary values?** (e.g. market, prices, tariffs, public prices, etc.)

**3. Background about the health system in your country:**

3.a) How is the publicly funded health care system financed in the country (e.g. general taxation, social security contributions). Is there a significant role for private insurance? Can people voluntarily opt-out of the public system?

3.b) How is the provision of publicly funded health care (hospitals, primary care, etc.) organized in the country (e.g. public providers, private providers or a mix).

3.c) How are providers in the publicly funded system reimbursed in the country (e.g. annual block grants / payment by activity)

**4. General information about the scope and purpose of the official or more widely used accounting system for healthcare services in your country**

4.a) How are the hospitals or healthcare providers sampled to be included in the accounting system or ad-hoc costing exercise? (e.g., all providers are included / only hospital providers / only publicly owned hospitals are included / only a sample of hospital providers)

4.b) How often is the costing exercise undertaken? (E.g. Annually? Ad-hoc?)

4.c) What is the primary purpose of the official costing exercise? (E.g. Estimating unit costs for economic evaluation? / Setting hospital tariffs or prices? / Benchmarking efficient providers?)

**5. Description of how the accounting system or ad hoc costing exercise classifies the outputs of the costing exercise (the cost objects)**

5.a) What system is used to categorize hospital inpatient and outpatient activity? (e.g. DRG / other)

5.b) What system is used to categorize primary care activity? (e.g. GP visits / other)

**6. Description of how the accounting system or ad hoc costing exercise identifies which resource items (healthcare inputs) are directly associated with the final outputs (the cost objects)**

6.a) Is the resource use for each cost object estimated in a very detailed way (micro-costing method or activity-based costing) or at a relatively aggregate way (gross-costing)?

**7. Description of how the value of each resource item or overhead is estimated**

7.a) Please describe how resource use items are valued (E.g. from published tariffs / from the hospital database / from standard unit costs, etc.)

7.b) Which variable and fixed overheads are included in the final cost object and how are they assigned to those cost objects

**8. Reporting variation and uncertainty**

8.a) What is the level of aggregation at which unit costs are available or published? (e.g., hospital level, region, national average, etc.)

8.b) Are only average (national) costs reported? Are the costs reported for each provider in the sample? Are measures of variation reported (e.g. IQR, range, SD)?

8.c) If average (national) costs are reported, how many institutions and/or observations are they based on? Is this information available in the respective databases?

8.d) How up to date are the costs published by the healthcare sector? (E.g. they refer to costs estimated during the previous financial year, or two years ago, or three years ago, etc.)

**Many thanks for your collaboration!**

GLOSSARY OF TERMS

| Terms | Definition in cost accounting | Explanation and example |
| --- | --- | --- |
| Direct costs | A cost that is used by a single cost object. | An expenditure that can be traced directly to a particular cost object. E.g., a high-cost pharmaceutical that is used for treating only a particular DRG and no other. |
| Indirect cost (variable overheads) | The cost of a resource that is used by more than one cost object, but varies with the quantity used. | E.g. Expenses which are recorded at departmental level which are shared between several patients, such as medical staff or nursing staff |
| General overheads (fixed overheads) | Expenses which are incurred at organisational level, do not vary with the number of patients treated, and are shared between several departments. | E.g. amortization of buildings, staff training costs, cost of water, electricity and heating |
| Top-down costing | A costing method where all costs the organization incurs over a given period are allocated to cost objects. | Direct costs are identified directly to cost objects. Indirect costs are ”apportioned” to cost objects. In full costing, both variable and fixed overheads will be apportioned to cost objects |
| Full-absorption costing | A top-down costing method. 100% of an organisations costs incurred over a given period are allocated to all the cost objects | Direct costs, variable overheads and fixed overheads are apportioned to cost objects. Sometimes required by financial reporting standards |
| Activity-based costing | A method of top-down micro-costing | Indirect expenditure is first allocated to tasks or activities, so that it can be apportioned to cost objects at a more detailed level of disaggregation than used in traditional top-down gross costing |
| Micro-costing | Costs are identified at very detailed level. | Drug costs, cost of surgeon, cost of nurse, material costs, amortization, capital costs, energies, etc. |
| Gross-costing | Costs are identified at highly aggregated level. | Inpatient costs, costs of department of traumatology, etc. |
| Cost object | Final product, process or service that are going to be costed. | E.g., Hospital GRDs. Normally in top-down methods, all final services performed by the organisation during the accounting period will be costed |
| Bottom-up | Cost components are valued by identifying resource use directly employed by each patient. | Patient-specific costs |
