# Supplementary material for: Description of day case costs and tariffs of cataract surgery from a sample of nine European countries
Source: Cost Eff Resour Alloc. 2022 Mar 5;20:11. doi: 10.1186/s12962-022-00346-3 (PMC8898401; doi:10.1186/s12962-022-00346-3)
Supplement: Supplementary file 2 — Additional file 2. Responses to questionnaire. [file 12962_2022_346_MOESM2_ESM.docx]

| **England** | **1. Official or frequently used sources of unit costs for EE** | <https://improvement.nhs.uk/resources/reference-costs/> |
| --- | --- | --- |
|  | **2. Are the unit costs in the former databases based on accounting costs of public or private health care institutions or on other types of monetary values?** | They are average unit costs from NHS trusts and NHS foundation trusts. |
|  | **3. Background about the health system in your country** | |
|  | How is the publicly funded health care system financed in the country? | Mainly through general taxation supplemented by National Insurance contributions. |
|  | Is there a significant role for private insurance? | Around 10.6% of the population. |
|  | Can people voluntarily opt-out of the public system? |  |
|  | How is the provision of publicly funded health care (hospitals, primary care, etc.) organized in the country (e.g. public providers, private providers or a mix)? | The large majority of health care expenditure has been on services provided by publicly owned (i.e. state owned) providers, including hospitals. the majority of NHS primary care in the UK is, and has been, provided by private individuals, partnerships and companies contracted to the NHS: GPs, dentists, pharmacists, opticians, etc. |
|  | How are providers in the publicly funded system reimbursed in the country (e.g. annual block grants / payment by activity)? | There is a mix system. HRGs are the basis of the national tariff for acute care in England, also known as ‘payment by results’. There are currently over 1,400 mandatory tariffs representing around 60% of payments made to hospitals and other acute providers. Many services such as mental health, some specialized services and community services remain outside the scope of tariff and they are still paid for through block contracts. |
|  | **4. General information about the scope and purpose of the official or more widely used accounting system for healthcare services in your country** | |
|  | How are the hospitals or healthcare providers sampled to be included in the accounting system or ad-hoc costing exercise? | All public providers are included. |
|  | How often is the costing exercise undertaken? | Annually. |
|  | What is the primary purpose of the official costing exercise? | To inform a number of work streams including the Model Hospital, the Getting It Right First Time (GIRFT) program and national tariff prices. |
|  | **5. Description of how the accounting system or ad hoc costing exercise classifies the outputs of the costing exercise (the cost objects)** | |
|  | What system is used to categorize hospital inpatient and outpatient activity? | Diagnosis-Related Groups that are in England known as Health resource groups (HRGs). |
|  | What system is used to categorize primary care activity? | N/A |
|  | **6. Description of how the accounting system or ad hoc costing exercise identifies which resource items (healthcare inputs) are directly associated with the final outputs (the cost objects)** | |
|  | Is the resource use for each cost object estimated in a very detailed way (micro-costing method or activity-based costing) or at a relatively aggregated way (gross-costing)? | Activity-based costing and micro costing. |
|  | **7. Description of how the value of each resource item or overhead is estimated** | |
|  | How are resource use items valued? | To determine the average cost for a specific intervention, the first step is to identify the relevant HRGs. The second step is to identify a weighted average cost from the total activity and costs across the required settings. Inpatient costs are split between those below the trim point (inlier) and those above the trim point (excess). When calculating a weighted average cost, the inlier and excess costs are summed but the excess bed day activity, which is already included in the inlier activity, is ignored. |
|  | Which variable and fixed overheads are included in the final cost object and how are they assigned to those cost objects? | All variable overheads are included. Research costs, teaching costs, financial costs and cost of depreciation of building are not included. |
|  | **8. Reporting variation and uncertainty** | |
|  | What is the level of aggregation at which unit costs are available or published? | National, regional and hospital level. |
|  | Are only average (national) costs reported? Are the costs reported for each provider in the sample? Are measures of variation reported? | The costs are reported for each provider in the sample. |
|  | If average (national) costs are reported, how many institutions and/or observations are they based on? Is this information available in the respective databases? | They are based on 232 NHS providers. This information is available for each year. |
|  | How up to date are the costs published by the healthcare sector? | Previous financial year. |

| **France** | **1. Official or frequently used sources of unit costs for EE** | <https://www.scansante.fr/applications/enc-mco>; <https://www.atih.sante.fr/tarifs-mco-et-had> |
| --- | --- | --- |
|  | **2. Are the unit costs in the former databases based on accounting costs of public or private health care institutions or on other types of monetary values?** | Accounting costs and tariffs |
|  | **3. Background about the health system in your country** | |
|  | How is the publicly funded health care system financed in the country? | - |
|  | Is there a significant role for private insurance? | - |
|  | Can people voluntarily opt-out of the public system? | - |
|  | How is the provision of publicly funded health care (hospitals, primary care, etc.) organized in the country (e.g. public providers, private providers or a mix)? | - |
|  | How are providers in the publicly funded system reimbursed in the country (e.g. annual block grants / payment by activity)? | - |
|  | **4. General information about the scope and purpose of the official or more widely used accounting system for healthcare services in your country** | |
|  | How are the hospitals or healthcare providers sampled to be included in the accounting system or ad-hoc costing exercise? | Voluntarily sample of public and private hospitals is included. |
|  | How often is the costing exercise undertaken? | Annually. |
|  | What is the primary purpose of the official costing exercise? | To inform national DRG tariffs for hospital reimbursement. |
|  | **5. Description of how the accounting system or ad hoc costing exercise classifies the outputs of the costing exercise (the cost objects)** | |
|  | What system is used to categorize hospital inpatient and outpatient activity? | Diagnosis-Related Groups |
|  | What system is used to categorize primary care activity? | - |
|  | **6. Description of how the accounting system or ad hoc costing exercise identifies which resource items (healthcare inputs) are directly associated with the final outputs (the cost objects)** | |
|  | Is the resource use for each cost object estimated in a very detailed way (micro-costing method or activity-based costing) or at a relatively aggregated way (gross-costing)? | Combination of micro-costing and gross-costing |
|  | **7. Description of how the value of each resource item or overhead is estimated** | |
|  | How are resource use items valued? | Combination of top-down and bottom-up |
|  | Which variable and fixed overheads are included in the final cost object and how are they assigned to those cost objects? | All variable overheads are included. Fixed overheads that are excluded are teaching costs, research costs and financial costs. |
|  | **8. Reporting variation and uncertainty** | |
|  | What is the level of aggregation at which unit costs are available or published? | Costs from production and tariffs from social health insurance point of view are published at national level. |
|  | Are only average (national) costs reported? Are the costs reported for each provider in the sample? Are measures of variation reported? | Only national averages. |
|  | If average (national) costs are reported, how many institutions and/or observations are they based on? Is this information available in the respective databases? | In 2020, 83 hospitals were included in the sample of public hospitals (for medical, surgical and obstetric wards). |
|  | How up to date are the costs published by the healthcare sector? | There is a two-year lag between the year of the data and the year of the application of prices in hospitals |

| **Germany** | **1. Official or frequently used sources of unit costs for EE** | There is no officially used source of unit costs for EE. |
| --- | --- | --- |
|  | **2. Are the unit costs in the former databases based on accounting costs of public or private health care institutions or on other types of monetary values?** | No. |
|  | **3. Background about the health system in your country** | |
|  | How is the publicly funded health care system financed in the country? | Social security contributions. |
|  | Is there a significant role for private insurance? | 10% of population (mainly civil servants, self-employed and very rich) and privately health insured. |
|  | Can people voluntarily opt-out of the public system? | Opt-out is possible for the previously mentioned groups. |
|  | How is the provision of publicly funded health care (hospitals, primary care, etc.) organized in the country (e.g. public providers, private providers or a mix)? | Mix of public providers (e.g. university clinics), private owners (e.g. private clinics or ambulatory practices) and non-for profit providers (e.g. church-owned hospitals). |
|  | How are providers in the publicly funded system reimbursed in the country (e.g. annual block grants / payment by activity)? | In hospitals: DRGs. |
|  | **4. General information about the scope and purpose of the official or more widely used accounting system for healthcare services in your country** | |
|  | How are the hospitals or healthcare providers sampled to be included in the accounting system or ad-hoc costing exercise? | Sample of hospital providers. |
|  | How often is the costing exercise undertaken? | Annually. |
|  | What is the primary purpose of the official costing exercise? | Calculating DRGs. |
|  | **5. Description of how the accounting system or ad hoc costing exercise classifies the outputs of the costing exercise (the cost objects)** | |
|  | What system is used to categorize hospital inpatient and outpatient activity? | Hospitals: DRGs; Outpatient: fee-for-service and lump-sums by patient |
|  | What system is used to categorize primary care activity? | fee-for-service and lump-sums by patient |
|  | **6. Description of how the accounting system or ad hoc costing exercise identifies which resource items (healthcare inputs) are directly associated with the final outputs (the cost objects)** | |
|  | Is the resource use for each cost object estimated in a very detailed way (micro-costing method or activity-based costing) or at a relatively aggregated way (gross-costing)? | DRGs: micro-costing in hospitals; general practitioners: relative aggregate |
|  | **7. Description of how the value of each resource item or overhead is estimated** | |
|  | How are resource use items valued? | Hospital database |
|  | Which variable and fixed overheads are included in the final cost object and how are they assigned to those cost objects? | Personnel costs are included by average time for the service. Nursing costs are excluded (financed separately). Among excluded fixed overheads are teaching costs, research costs, depreciation of building and financial costs. |
|  | **8. Reporting variation and uncertainty** | |
|  | What is the level of aggregation at which unit costs are available or published? | National average. |
|  | Are only average (national) costs reported? Are the costs reported for each provider in the sample? Are measures of variation reported? | Only national averages. |
|  | If average (national) costs are reported, how many institutions and/or observations are they based on? Is this information available in the respective databases? | 217 hospitals (3.7 million cases) available in the DRG catalogue |
|  | How up to date are the costs published by the healthcare sector? | They refer to the previous year. |

| **Italy** | **1. Official or frequently used sources of unit costs for EE** | Outpatient specialist care: <https://www.trovanorme.salute.gov.it/norme/renderPdf.spring?seriegu=SG&datagu=28/01/2013&redaz=13A00528&artp=3&art=1&subart=1&subart1=10&vers=1&prog=001>;  hospital care: <https://www.trovanorme.salute.gov.it/norme/renderPdf.spring?seriegu=SG&datagu=28/01/2013&redaz=13A00528&artp=1&art=1&subart=1&subart1=10&vers=1&prog=001> |
| --- | --- | --- |
|  | **2. Are the unit costs in the former databases based on accounting costs of public or private health care institutions or on other types of monetary values?** | They are tariffs. |
|  | **3. Background about the health system in your country** | |
|  | How is the publicly funded health care system financed in the country? | By taxes. |
|  | Is there a significant role for private insurance? | Around 14 million citizens had a private insurance in 2017. |
|  | Can people voluntarily opt-out of the public system? | No, but they can opt for the “free-market” for single interventions. |
|  | How is the provision of publicly funded health care (hospitals, primary care, etc.) organized in the country (e.g. public providers, private providers or a mix)? | Hospitals and specialist outpatient care is provided by a mix of public and accredited private providers. Primary care is provided by general practitioners affiliated with Italian National Health Service. |
|  | How are providers in the publicly funded system reimbursed in the country (e.g. annual block grants / payment by activity)? | The reimbursement is based on tariffs established in advance. |
|  | **4. General information about the scope and purpose of the official or more widely used accounting system for healthcare services in your country** | |
|  | How are the hospitals or healthcare providers sampled to be included in the accounting system or ad-hoc costing exercise? | A sample of 41 (public or private) healthcare providers were selected according to the criteria of efficiency, efficacy, quality and appropriateness of care, plus the availability of robust cost data. |
|  | How often is the costing exercise undertaken? | Irregularly (last costing exercise dates back 2011). |
|  | What is the primary purpose of the official costing exercise? | Setting hospital and outpatient tariffs. |
|  | **5. Description of how the accounting system or ad hoc costing exercise classifies the outputs of the costing exercise (the cost objects)** | |
|  | What system is used to categorize hospital inpatient and outpatient activity? | Hospital: Diagnosis-Related Groups. Outpatient activity is categorized using alphanumeric codes derived from the Italian version of the international Classification of Diseases ICD9-CM. |
|  | What system is used to categorize primary care activity? | There is no classification system for primary care visits. |
|  | **6. Description of how the accounting system or ad hoc costing exercise identifies which resource items (healthcare inputs) are directly associated with the final outputs (the cost objects)** | |
|  | Is the resource use for each cost object estimated in a very detailed way (micro-costing method or activity-based costing) or at a relatively aggregated way (gross-costing)? | It depends on each study, however, gross-costing prevails. |
|  | **7. Description of how the value of each resource item or overhead is estimated** | |
|  | How are resource use items valued? | The legislation (D.lgs. 502/92) imposes a linkage between tariffs and standard costs. |
|  | Which variable and fixed overheads are included in the final cost object and how are they assigned to those cost objects? | All variable overheads are included. Teaching costs, research costs and financial costs are excluded. |
|  | **8. Reporting variation and uncertainty** | |
|  | What is the level of aggregation at which unit costs are available or published? | National tariffs. |
|  | Are only average (national) costs reported? Are the costs reported for each provider in the sample? Are measures of variation reported? | Some regions publish their tariffs (e.g. Lazio). |
|  | If average (national) costs are reported, how many institutions and/or observations are they based on? Is this information available in the respective databases? | 41 providers |
|  | How up to date are the costs published by the healthcare sector? | The most recent year of inpatient DRGs date back 2012. |

| **Poland** | **1. Official or frequently used sources of unit costs for EE** | <http://www.nfz.gov.pl/zarzadzenia-prezesa/zarzadzenia-prezesa-nfz/> |
| --- | --- | --- |
|  | **2. Are the unit costs in the former databases based on accounting costs of public or private health care institutions or on other types of monetary values?** | National tariffs in Poland are based on data provided by all or selected healthcare providers taking into account volume of the services. A health care provider may have the contract with National Health Fund (NHF) for particular healthcare services depending on whether the provider is private or public. Healthcare providers are selected in a competition based on predefined criteria. |
|  | **3. Background about the health system in your country** | |
|  | How is the publicly funded health care system financed in the country? | The Polish health care is based on a general health insurance system. The reimbursed health services are provided to Polish residents who are covered by the general health insurance. This can be either on a compulsory or a voluntary basis. Compulsory health insurance covers 98% of the population. The health insurance contribution is paid by the employer (in case of self-employment as well). |
|  | Is there a significant role for private insurance? | Private expenditure (estimated for 30% of the total health expenditure) is mainly out-of-pocket payment with minor supplemental role of private insurance and quasi-insurance. |
|  | Can people voluntarily opt-out of the public system? | No. |
|  | How is the provision of publicly funded health care (hospitals, primary care, etc.) organized in the country (e.g. public providers, private providers or a mix)? | There are public and private providers who might have contract with the NHF. Private providers are more common on a lower level of care. Private hospitals in general belong to a bigger network of healthcare providers and do not have contracts with NHF. |
|  | How are providers in the publicly funded system reimbursed in the country (e.g. annual block grants / payment by activity)? | They are funded on the basis of the contracts concluded with the payer for a year or several years. Settlements are made each month. They are also made based on activity (except for capitation-based model, e.g. primary health care). |
|  | **4. General information about the scope and purpose of the official or more widely used accounting system for healthcare services in your country** | |
|  | How are the hospitals or healthcare providers sampled to be included in the accounting system or ad-hoc costing exercise? | Only providers that have contract with the payer are included. If there are only a few such providers, all of them are invited to take part. If there are many providers, the sampled ones providing a specific service are selected randomly. |
|  | How often is the costing exercise undertaken? | Annually. |
|  | What is the primary purpose of the official costing exercise? | Setting national tariffs. |
|  | **5. Description of how the accounting system or ad hoc costing exercise classifies the outputs of the costing exercise (the cost objects)** | |
|  | What system is used to categorize hospital inpatient and outpatient activity? | Diagnosis-Related Groups. |
|  | What system is used to categorize primary care activity? | Capitation method. |
|  | **6. Description of how the accounting system or ad hoc costing exercise identifies which resource items (healthcare inputs) are directly associated with the final outputs (the cost objects)** | |
|  | Is the resource use for each cost object estimated in a very detailed way (micro-costing method or activity-based costing) or at a relatively aggregated way (gross-costing)? | If it is justified (in case of very expensive services) and possible (due to data availability), the micro-costing method is used. In other cases, the gross-costing approach is used. |
|  | **7. Description of how the value of each resource item or overhead is estimated** | |
|  | How are resource use items valued? | Resource items are valued based on the hospital database. They are also valued using the official prices of drugs. Sometimes tariffs of the procedures are used as well. |
|  | Which variable and fixed overheads are included in the final cost object and how are they assigned to those cost objects? | All costs reported by the hospital are included in the valuation. Excluded fixed overheads are teaching and research costs. |
|  | **8. Reporting variation and uncertainty** | |
|  | What is the level of aggregation at which unit costs are available or published? | National tariffs. |
|  | Are only average (national) costs reported? Are the costs reported for each provider in the sample? Are measures of variation reported? | The document describing the process of setting tariffs presents the share of specific cost components in the total cost of the service (i.e. hospitalization, medical devices, medicinal products and procedures). Before the tariff is set, in the course of consultations the results of sensitivity analyses are presented. In those analyses, the variables with the largest spread of costs are tested. |
|  | If average (national) costs are reported, how many institutions and/or observations are they based on? Is this information available in the respective databases? | The goal is to create a database with at least 10% of services and service providers. However, it is not always successful. |
|  | How up to date are the costs published by the healthcare sector? | The data is historical, usually coming from the previous year. |

| **Portugal** | **1. Official or frequently used sources of unit costs for EE** | <http://www.acss.min-saude.pt/category/acss_pt/tabelas-e-impressos/> |
| --- | --- | --- |
|  | **2. Are the unit costs in the former databases based on accounting costs of public or private health care institutions or on other types of monetary values?** | Regarding DRG prices, these make use of accounting costs taken from the Portuguese hospital costs database –which considers annual public hospitals cost information– and of Maryland cost weights. |
|  | **3. Background about the health system in your country** | |
|  | How is the publicly funded health care system financed in the country? | General taxation. |
|  | Is there a significant role for private insurance? | Private health insurance has a non-negligible size in the Portuguese health care system, but it mostly assumes a complementary and supplementary nature to the NHS |
|  | Can people voluntarily opt-out of the public system? | Although legally it has been possible to opt-out of the public system, there has been no opting-out in practice. |
|  | How is the provision of publicly funded health care (hospitals, primary care, etc.) organized in the country (e.g. public providers, private providers or a mix)? | It is mostly provided by public hospitals and public primary care centers. A few hospitals operate as public-private partnerships. |
|  | How are providers in the publicly funded system reimbursed in the country (e.g. annual block grants / payment by activity)? | NHS hospitals budgets are defined through annual agreements with the Ministry of Health that define production quantities and prices. Further to quantities and prices for several production lines (including inpatient, outpatient, consultations, day surgery), the agreements entail performance incentives, penalties, regional adjustments, specific autonomous funding for some types of care, R&D adjustments. |
|  | **4. General information about the scope and purpose of the official or more widely used accounting system for healthcare services in your country** | |
|  | How are the hospitals or healthcare providers sampled to be included in the accounting system or ad-hoc costing exercise? | All NHS – publicly owned hospitals – are within the scope of the accounting system and report their activity and costs annually to the Central Administration of Health Services (ACSS). |
|  | How often is the costing exercise undertaken? | Annually. |
|  | What is the primary purpose of the official costing exercise? |  |
|  | **5. Description of how the accounting system or ad hoc costing exercise classifies the outputs of the costing exercise (the cost objects)** | |
|  | What system is used to categorize hospital inpatient and outpatient activity? |  |
|  | What system is used to categorize primary care activity? |  |
|  | **6. Description of how the accounting system or ad hoc costing exercise identifies which resource items (healthcare inputs) are directly associated with the final outputs (the cost objects)** | |
|  | Is the resource use for each cost object estimated in a very detailed way (micro-costing method or activity-based costing) or at a relatively aggregated way (gross-costing)? |  |
|  | **7. Description of how the value of each resource item or overhead is estimated** | |
|  | How are resource use items valued? |  |
|  | Which variable and fixed overheads are included in the final cost object and how are they assigned to those cost objects? |  |
|  | **8. Reporting variation and uncertainty** | |
|  | What is the level of aggregation at which unit costs are available or published? |  |
|  | Are only average (national) costs reported? Are the costs reported for each provider in the sample? Are measures of variation reported? |  |
|  | If average (national) costs are reported, how many institutions and/or observations are they based on? Is this information available in the respective databases? |  |
|  | How up to date are the costs published by the healthcare sector? |  |

| **Slovenia** | **1. Official or frequently used sources of unit costs for EE** |  |
| --- | --- | --- |
|  | **2. Are the unit costs in the former databases based on accounting costs of public or private health care institutions or on other types of monetary values?** |  |
|  | **3. Background about the health system in your country** | |
|  | How is the publicly funded health care system financed in the country? |  |
|  | Is there a significant role for private insurance? |  |
|  | Can people voluntarily opt-out of the public system? |  |
|  | How is the provision of publicly funded health care (hospitals, primary care, etc.) organized in the country (e.g. public providers, private providers or a mix)? |  |
|  | How are providers in the publicly funded system reimbursed in the country (e.g. annual block grants / payment by activity)? |  |
|  | **4. General information about the scope and purpose of the official or more widely used accounting system for healthcare services in your country** | |
|  | How are the hospitals or healthcare providers sampled to be included in the accounting system or ad-hoc costing exercise? |  |
|  | How often is the costing exercise undertaken? |  |
|  | What is the primary purpose of the official costing exercise? |  |
|  | **5. Description of how the accounting system or ad hoc costing exercise classifies the outputs of the costing exercise (the cost objects)** | |
|  | What system is used to categorize hospital inpatient and outpatient activity? |  |
|  | What system is used to categorize primary care activity? |  |
|  | **6. Description of how the accounting system or ad hoc costing exercise identifies which resource items (healthcare inputs) are directly associated with the final outputs (the cost objects)** | |
|  | Is the resource use for each cost object estimated in a very detailed way (micro-costing method or activity-based costing) or at a relatively aggregated way (gross-costing)? |  |
|  | **7. Description of how the value of each resource item or overhead is estimated** | |
|  | How are resource use items valued? |  |
|  | Which variable and fixed overheads are included in the final cost object and how are they assigned to those cost objects? |  |
|  | **8. Reporting variation and uncertainty** | |
|  | What is the level of aggregation at which unit costs are available or published? |  |
|  | Are only average (national) costs reported? Are the costs reported for each provider in the sample? Are measures of variation reported? |  |
|  | If average (national) costs are reported, how many institutions and/or observations are they based on? Is this information available in the respective databases? |  |
|  | How up to date are the costs published by the healthcare sector? |  |

| **Spain** | **1. Official or frequently used sources of unit costs for EE** |  |
| --- | --- | --- |
|  | **2. Are the unit costs in the former databases based on accounting costs of public or private health care institutions or on other types of monetary values?** |  |
|  | **3. Background about the health system in your country** | |
|  | How is the publicly funded health care system financed in the country? |  |
|  | Is there a significant role for private insurance? |  |
|  | Can people voluntarily opt-out of the public system? |  |
|  | How is the provision of publicly funded health care (hospitals, primary care, etc.) organized in the country (e.g. public providers, private providers or a mix)? |  |
|  | How are providers in the publicly funded system reimbursed in the country (e.g. annual block grants / payment by activity)? |  |
|  | **4. General information about the scope and purpose of the official or more widely used accounting system for healthcare services in your country** | |
|  | How are the hospitals or healthcare providers sampled to be included in the accounting system or ad-hoc costing exercise? |  |
|  | How often is the costing exercise undertaken? |  |
|  | What is the primary purpose of the official costing exercise? |  |
|  | **5. Description of how the accounting system or ad hoc costing exercise classifies the outputs of the costing exercise (the cost objects)** | |
|  | What system is used to categorize hospital inpatient and outpatient activity? |  |
|  | What system is used to categorize primary care activity? |  |
|  | **6. Description of how the accounting system or ad hoc costing exercise identifies which resource items (healthcare inputs) are directly associated with the final outputs (the cost objects)** | |
|  | Is the resource use for each cost object estimated in a very detailed way (micro-costing method or activity-based costing) or at a relatively aggregated way (gross-costing)? |  |
|  | **7. Description of how the value of each resource item or overhead is estimated** | |
|  | How are resource use items valued? |  |
|  | Which variable and fixed overheads are included in the final cost object and how are they assigned to those cost objects? |  |
|  | **8. Reporting variation and uncertainty** | |
|  | What is the level of aggregation at which unit costs are available or published? |  |
|  | Are only average (national) costs reported? Are the costs reported for each provider in the sample? Are measures of variation reported? |  |
|  | If average (national) costs are reported, how many institutions and/or observations are they based on? Is this information available in the respective databases? |  |
|  | How up to date are the costs published by the healthcare sector? |  |

| **Sweden** | **1. Official or frequently used sources of unit costs for EE** |  |
| --- | --- | --- |
|  | **2. Are the unit costs in the former databases based on accounting costs of public or private health care institutions or on other types of monetary values?** |  |
|  | **3. Background about the health system in your country** | |
|  | How is the publicly funded health care system financed in the country? |  |
|  | Is there a significant role for private insurance? |  |
|  | Can people voluntarily opt-out of the public system? |  |
|  | How is the provision of publicly funded health care (hospitals, primary care, etc.) organized in the country (e.g. public providers, private providers or a mix)? |  |
|  | How are providers in the publicly funded system reimbursed in the country (e.g. annual block grants / payment by activity)? |  |
|  | **4. General information about the scope and purpose of the official or more widely used accounting system for healthcare services in your country** | |
|  | How are the hospitals or healthcare providers sampled to be included in the accounting system or ad-hoc costing exercise? |  |
|  | How often is the costing exercise undertaken? |  |
|  | What is the primary purpose of the official costing exercise? |  |
|  | **5. Description of how the accounting system or ad hoc costing exercise classifies the outputs of the costing exercise (the cost objects)** | |
|  | What system is used to categorize hospital inpatient and outpatient activity? |  |
|  | What system is used to categorize primary care activity? |  |
|  | **6. Description of how the accounting system or ad hoc costing exercise identifies which resource items (healthcare inputs) are directly associated with the final outputs (the cost objects)** | |
|  | Is the resource use for each cost object estimated in a very detailed way (micro-costing method or activity-based costing) or at a relatively aggregated way (gross-costing)? |  |
|  | **7. Description of how the value of each resource item or overhead is estimated** | |
|  | How are resource use items valued? |  |
|  | Which variable and fixed overheads are included in the final cost object and how are they assigned to those cost objects? |  |
|  | **8. Reporting variation and uncertainty** | |
|  | What is the level of aggregation at which unit costs are available or published? |  |
|  | Are only average (national) costs reported? Are the costs reported for each provider in the sample? Are measures of variation reported? |  |
|  | If average (national) costs are reported, how many institutions and/or observations are they based on? Is this information available in the respective databases? |  |
|  | How up to date are the costs published by the healthcare sector? |  |
