# Supplementary material for: Description of day case costs and tariffs of cataract surgery from a sample of nine European countries
Source: Cost Eff Resour Alloc. 2022 Mar 5;20:11. doi: 10.1186/s12962-022-00346-3 (PMC8898401; doi:10.1186/s12962-022-00346-3)
Supplement: Supplementary file 3 — Additional file 3. PRISMA flow diagram. [file 12962_2022_346_MOESM3_ESM.docx]

**Studies identified via databases**

Records identified from PubMed and Scopus: (n = 54)

**Identification**

Records excluded (n = 48)

Not related with cost or cataract surgery

Records screened (n = 54)

**Screening**

Reports excluded: Cost source not identified (n = 1)

Reports assessed for eligibility

(n = 6)

Studies included in review (n = 5)

**Included**

From: Page MJ, McKenzie JE, Bossuyt PM, Boutron I, Hoffmann TC, Mulrow CD, et al. The PRISMA 2020 statement: an updated guideline for reporting systematic reviews. BMJ 2021;372:n71. doi: 10.1136/bmj.n71

Notes: The search was completed with an additional search on Google Scholar and reference lists of relevant studies identified from the search were also reviewed.
